# Harnessing cellular immunotherapy for cholangiocarcinoma: an integrated roadmap for overcoming resistance

**DOI:** 10.3389/fimmu.2026.1757504

**Published:** 2026-05-08

**Authors:** Saied Froghi, Astero Klampatsa, Brian Davidson

**Affiliations:** 1 Division of Surgery & Interventional Sciences, Royal Free Hospital, University College London, London, United Kingdom; 2 Thoracic Oncology Immunotherapy Group, Division of Cancer Biology, Institute of Cancer Research, Sutton, United Kingdom

**Keywords:** ACT, adoptive cell therapy, armoured cars, CCA, cholangiocarcinoma, combination immunotherapy, immune checkpoints, TME

## Abstract

The rising global incidence and dismal prognosis of cholangiocarcinoma (CCA) underscore the profound limitations of standard therapies. While chimeric antigen receptor (CAR)-based cellular immunotherapies represent a paradigm shift in oncology, their success in CCA is fundamentally constrained by a desmoplastic, immunosuppressive tumour microenvironment (TME) and significant tumour antigen heterogeneity. This review advances the thesis that overcoming these barriers requires an integrated approach combining multi-antigen, armoured CAR designs with rational adjuvant strategies (i.e combination therapy). We provide a comparative analysis of key tumour-associated antigens (TAAs)-including MUC1, c-MET, and the cancer stem cell marker CD133-evaluating their expression profiles, preclinical efficacy, and clinical status. The review further deconstructs the core mechanisms of therapeutic resistance in CCA-spanning physical, immunological, and metabolic barriers-and map them to next-generation engineering strategies designed to counteract them. In a novel synthesis, we explore the synergistic potential of combining CAR therapies with checkpoint inhibitors and immunomodulatory natural compounds. Critically appraising the current clinical trial landscape, we identify key weaknesses and propose strategic recommendations for biomarker-driven, adaptive trial designs. Finally, we present a forward-looking, four-pillar roadmap for future research, positioning the integration of advanced CAR engineering, multi-antigen platforms, synergistic adjuvants, and alternative effectors as the definitive research agenda for translating the promise of cellular immunotherapy into a clinical reality for CCA.

## 1 Introduction

Cholangiocarcinoma (CCA), an aggressive malignancy of the biliary tract, represents a growing global health challenge with a persistently high mortality rate (1–3). The failure of standard treatments-including surgery, chemotherapy, and radiation-to provide curative treatment or durable responses for many patients highlights an urgent, unmet clinical need (4–6). More recently, immunotherapies based on immune checkpoint inhibitors, while transformative in other cancers, have yielded only modest benefits in advanced CCA. In previously treated disease, single-agent PD-1 blockade has demonstrated objective response rates (ORR) of approximately 5–13%, with median overall survival (OS) typically ranging from 7 to 9 months in unselected populations (7). The addition of durvalumab to gemcitabine–cisplatin in the TOPAZ-1 trial improved median OS from 11.5 to 12.9 months, with a 24-month survival rate of 24.9% vs. 10.4% for chemotherapy alone, establishing chemo-immunotherapy as a new first-line standard (8, 9). However, durable responses remain limited to a minority of patients, largely due to its non-T-cell inflamed, immunosuppressive tumour microenvironment (TME) characteristic of CCA (10–13).

Cellular therapies, particularly those using chimeric antigen receptor (CAR)-engineered T cells and Natural Killer (NK) cells, offer a paradigm shift (14–19). By redirecting immune effectors to target tumour-associated antigens (TAAs) directly and independent of the major histocompatibility complex (MHC), they have the potential to overcome the limitations of conventional treatments (14, 20–23). However, early efforts in CCA have been met with formidable biological resistance (16, 24). In CCA, these challenges are magnified by its fibrotic, immune-excluded stroma and paucity of tumour-specific antigens. Nonetheless, the emergence of new molecular targets-such as Claudin 18.2, MUC1, HER2, and EGFR-has renewed interest in adoptive cell therapy for CCA.

This review advances the core thesis that overcoming antigen heterogeneity and the immunosuppressive TME in CCA requires integrated multi-antigen, armoured CAR designs combined with rational synergistic strategies. By synthesizing preclinical innovations, critically appraising the clinical landscape, and proposing a strategic roadmap, we aim to define the future research agenda for cellular immunotherapy in cholangiocarcinoma.

## 2 Comparative landscape of actionable targets in CCA

The foundation of effective CAR therapy rests on identifying and validating suitable TAAs (25). The ideal target exhibits high, uniform expression on malignant cells, minimal presence on healthy tissues, functional relevance to tumour biology, and accessibility on the cell surface. While no perfect antigen exists in CCA, a comparative synthesis of preclinical and clinical evidence has identified several high-priority candidates with distinct biological rationales and translational potential. Here, we provide a systematic, antigen-by-antigen analysis that contextualizes expression patterns, CCA-specific challenges, and the current evidence base supporting their therapeutic pursuit. While the provided sources do not contain sufficient pooled cohort data to perform a formal meta-analysis of antigen prevalence, a comparative synthesis of the existing literature reveals several high-priority candidates, each with a distinct profile of opportunities and risks is summarised in **Table 1**.

**Table 1:** Comparative analysis of key antigen targets for CAR-based therapy in cholangiocarcinoma.

Target antigen	Expression profile in CCA	Advantages & preclinical efficacy highlights	Limitations & toxicity risks	Clinical status
MUC1	Highly expressed (50-86.5% of tissues); associated with poor prognosis.	Tumour-specific hypoglycosylated forms allow for high specificity. 4th-gen CAR-T cells show potent lysis (~66%) and disrupt 3D spheroids.	Low risk when targeting tumour-specific glycoforms.	Multiple Phase I/II trials ongoing for solid tumours (e.g., NCT04025216, NCT05239143) (26, 27).
c-MET	High expression in up to 91.3% of tissues; correlates with poor prognosis and shorter survival.	Strong CAR-NK cell-mediated killing demonstrated; efficacy correlates with expression level.	On-target, off-tumour toxicity is a concern as c-MET is found in some adjacent normal tissues.	Preclinical; no published trial results for CCA (14, 15, 28–30).
CD133	Cancer Stem Cell (CSC) marker; high expression in ~81% of CCA tissues. Associated with progression and recurrence.	Targets the root of tumour recurrence. 4th-gen CAR-T cells show potent cytotoxicity (~58%) and cytokine production.	Potential risk of targeting normal stem or progenitor cells that may express CD133.	Phase I trial (NCT02541370) reported a 4.5-month PR in a single patient as part of cocktail therapy (31).
EGFR	Abundant expression reported in CCA tumour tissues.	Clinical activity demonstrated with both CR and PRs reported.	High risk of on-target, off-tumour toxicity (skin, pulmonary, endothelial) due to widespread normal tissue expression.	Multiple Phase I trials completed; 1 CR, 10 SD (NCT01869166) and 4 PR, 8 SD reported (32).
HER2	Expressed in a subset of intrahepatic and extrahepatic CCA.	Validated target in other solid tumours.	Known risk of cardiotoxicity if not carefully managed.	Phase I study (NCT01935843) in BTCs/pancreatic cancers showed 1 PR, 5 SD (28).
CLDN18.2	Expression in CCA makes it a promising candidate, validated in other GI cancers.	High specificity as normal-tissue expression is limited to gastric mucosa tight junctions.	Low toxicity reported in clinical trials for gastric cancer.	Phase I trials (NCT03159819, NCT03874897) ongoing/completed; responses observed in GI cancers (33, 34).

### 2.1 MUC1: a high-frequency target with clinical precedent

**Figure 1:**
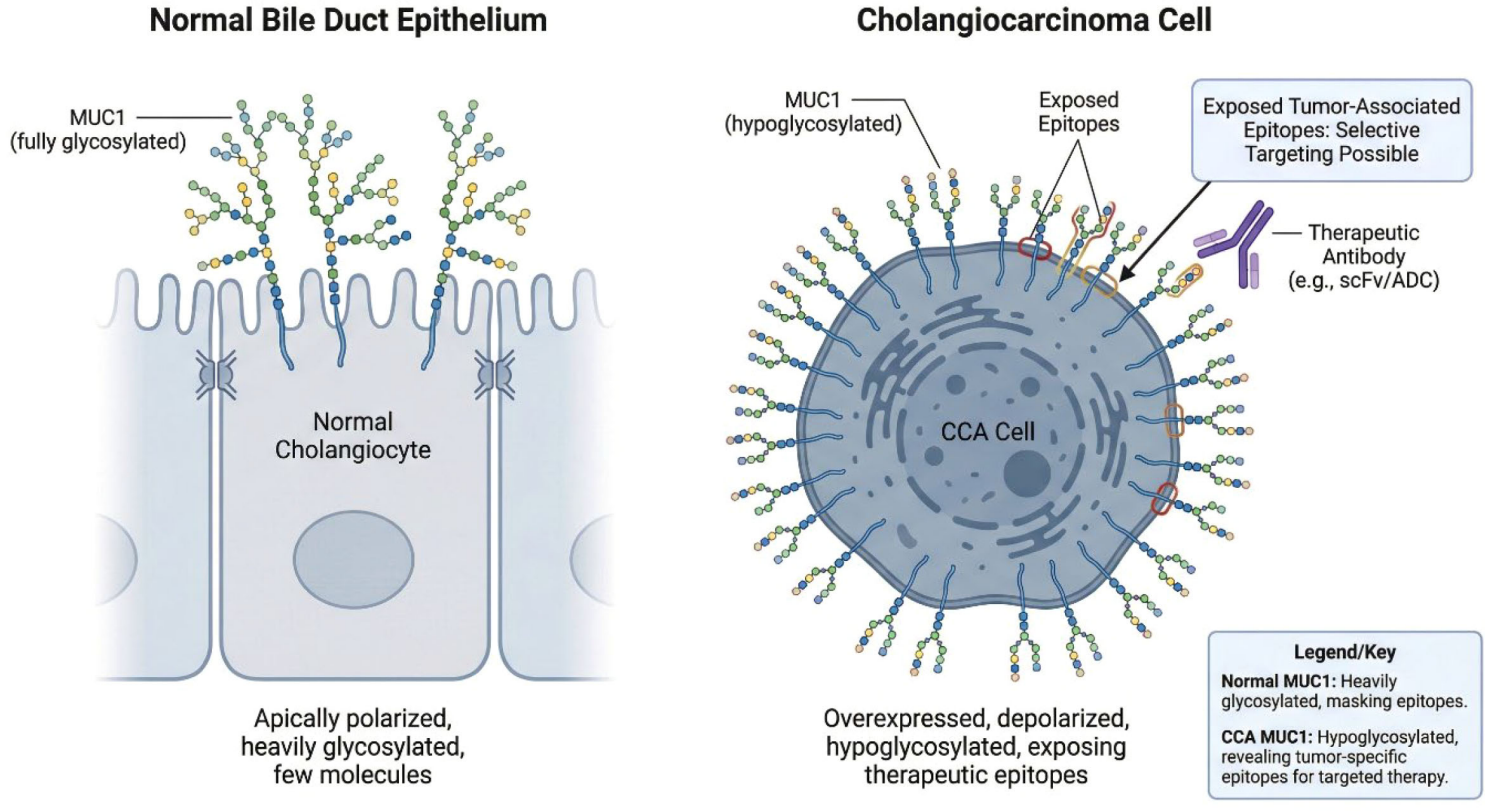
Differential MUC1 expression and glycosylation in normal bile duct epithelium versus CCA. In normal cholangiocytes, MUC1 is apically polarized and heavily glycosylated, limiting exposure of underlying peptide epitopes. In contrast, CCA cells exhibit overexpression, loss of polarity, and hypoglycosylation of MUC1, resulting in the exposure of tumour-associated epitopes. These cancer-specific glycoforms create a therapeutic window for selective targeting by antibodies, antibody–drug conjugates (ADCs), or CAR-based strategies.

Mucin 1 (MUC1) (**Figure 1**) is aberrantly overexpressed in approximately 60–80% of CCA cases, with significantly higher levels than in adjacent normal bile duct epithelium (16, 35). Malignant transformation is associated with loss of epithelial polarity and hypoglycosylation, exposing tumour-associated epitopes that create a therapeutic window for selective targeting. In preclinical studies specifically in CCA models, fourth-generation anti-MUC1 CAR-T cells demonstrated potent cytotoxicity (66% lysis of KKU-213A CCA cells at 5:1 E:T ratio) and effective disruption of three-dimensional tumour spheroids (36). These anti-MUC1-CAR4 T cells produced increased levels of TNF-α, IFN-γ, and granzyme B when exposed to MUC1-expressing CCA cells, while showing negligible activity against immortalized cholangiocytes (36).

#### 2.1.1 Clinical relevance in CCA

MUC1 expression correlates with advanced stage, lymph node metastasis, and poorer prognosis (37, 38), suggesting that MUC1-high tumours represent an aggressive biological subset that could benefit from intensive immunotherapy. Given the established safety profile and moderate clinical activity, MUC1 remains a rational backbone for multi-antigen CAR strategies.

While MUC1-directed CAR-T therapy has entered clinical trials for multiple solid tumours (i.e HCC, pancreatic, breast tumours) (NCT02587689, NCT04020575, NCT04025216), published results specifically in CCA patients remain unavailable (26, 39). The principal challenge with MUC1 targeting lies in its expression on normal epithelia, necessitating CAR designs optimized for tumour-specific glycoform recognition.

### 2.2 c-MET: exploiting receptor tyrosine kinase addiction

**Figure 2:**
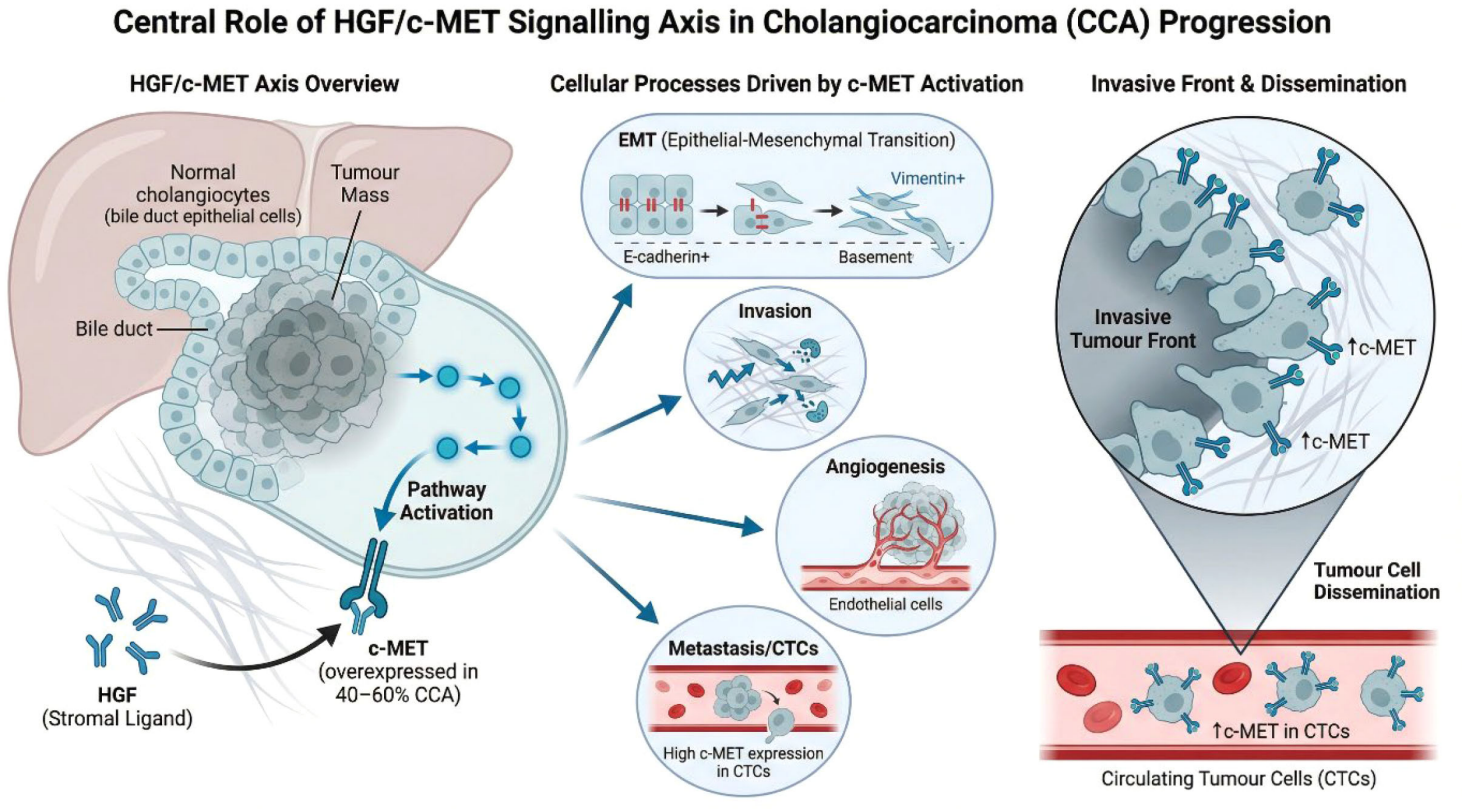
Central role of the HGF/c-MET signalling axis in CCA progression. Hepatocyte growth factor (HGF), produced by stromal components, binds to the c-MET receptor, which is overexpressed in approximately 40–60% of CCA cases. Activation of c-MET triggers downstream signalling pathways (including MEK/MAPK), driving epithelial–mesenchymal transition (EMT), invasion, angiogenesis, and metastatic dissemination. c-MET expression is enriched at the invasive tumour front and in circulating tumour cells (CTCs), highlighting its functional role in tumour progression and systemic spread.

The hepatocyte growth factor (HGF)/c-MET signalling axis plays a central role in CCA pathogenesis and progression (**Figure 2**) (40). Immunohistochemical analyses demonstrate c-MET protein expression in 45.0% of intrahepatic CCA and 68.4% of extrahepatic CCA cases, with high-level overexpression observed in 11.7% and 16.2%, respectively (41, 42). Molecular profiling further identifies c-MET gene amplification in a substantial subset of intrahepatic CCA, including 15.8% with high-frequency amplification (c-MET/CEP7 ratio >4.0) and 30.8% with low-frequency amplification, with high-level amplification strongly correlating with protein overexpression and aggressive clinicopathologic features (42).

Functionally, HGF-mediated activation of c-MET triggers MEK/MAPK signalling, promoting epithelial–mesenchymal transition (EMT), invasion, angiogenesis, and resistance to apoptosis (40, 43). Inhibition of c-MET signalling using small interfering RNA or MEK blockade suppresses HGF-induced invasion in CCA cell lines, confirming its mechanistic role in tumour aggressiveness (43).

#### 2.2.1 Clinical relevance in CCA

c-MET high expression is significantly associated with poor prognosis and reduced 5-year survival in both intrahepatic CCA (p=0.0013) and overall CCA cohorts (p=0.0046) (41). High-frequency amplification defines a more aggressive molecular subset and represents a potential biomarker for patient stratification (42). Given its direct contribution to invasion and metastasis, c-MET remains a biologically rational therapeutic target in advanced CCA, although CAR-T strategies targeting c-MET have not yet entered clinical evaluation and remain a future translational opportunity (43).

### 2.3 CD133: targeting the cancer stem cell niche

CD133 (prominin-1) marks a cancer stem-like cell (CSC) subpopulation in CCA that exhibits disproportionate tumorigenic capacity and aggressive biological behaviour (44, 45). Immunohistochemical studies demonstrate CD133 expression in 48-68% of CCA specimens, with expression patterns varying by tumour differentiation status (44–46). CD133-positive cells isolated from CCA cell lines display enhanced invasive capacity compared to CD133-depleted populations, supporting their role in metastatic progression (44).

The prognostic significance of CD133 expression in CCA remains complex and somewhat controversial. Shimada et al. reported that CD133-positive intrahepatic CCA patients had markedly inferior outcomes, with 5-year survival rates of 8.0% compared to 57.0% in CD133-negative patients (45). This finding was corroborated by Leelawat et al., who demonstrated that strong CD133 expression (>50% of cells) was significantly associated with lymph node metastasis (p=0.009) and positive surgical margins (p=0.011) (44). However, some studies have reported opposite findings, with CD133 expression correlating with better differentiation and improved prognosis (46), highlighting tumour heterogeneity and the need for standardized assessment criteria.

#### 2.3.1 Clinical translation of CD133-directed CAR-T therapy

A case report by Feng et al. described sequential treatment with EGFR-CAR-T followed by CD133-CAR-T cells in a 52-year-old woman with advanced metastatic CCA who had failed chemotherapy and radiotherapy (47). The patient achieved an 8.5-month partial response following EGFR-CAR-T therapy and a subsequent 4.5-month partial response with CD133-CAR-T treatment. However, the therapy was associated with significant toxicity, including skin rashes with epidermal loss and vascular damage (47). A subsequent Phase I/II trial evaluating CD133-CAR-T cells across multiple solid tumours included one CCA patient who developed Grade 3 cytokine release syndrome and skin/mucosal vasculature damage but achieved a 4.5-month partial remission (48).

#### 2.3.2 Safety considerations

Although CD133 is expressed on normal stem cells (hematopoietic, intestinal, neural progenitors), the limited clinical experience to date has not revealed dose-limiting hematologic or neurologic toxicity in the small number of patients treated (48). However, dermatologic and vascular toxicities have been observed, and long-term safety data remain limited.

#### 2.3.3 Clinical relevance in CCA

Elevated CD133 expression correlates with aggressive disease features including lymph node metastasis, positive surgical margins, and intrahepatic metastasis (44, 45). When present, CD133 positivity identifies a subset of patients with significantly worse prognosis who may benefit from stem-cell-directed therapeutic strategies, though further clinical validation is required to establish CD133-targeted CAR-T therapy as a viable treatment approach for CCA. An ongoing Phase I/II dose-escalation trial (NCT02541370) is aimed at evaluating CD133-CAR-T cells across multiple CD133-positive malignancies including cholangiocarcinoma (31).

### 2.4 EGFR: leveraging growth factor dependence

**Figure 3:**
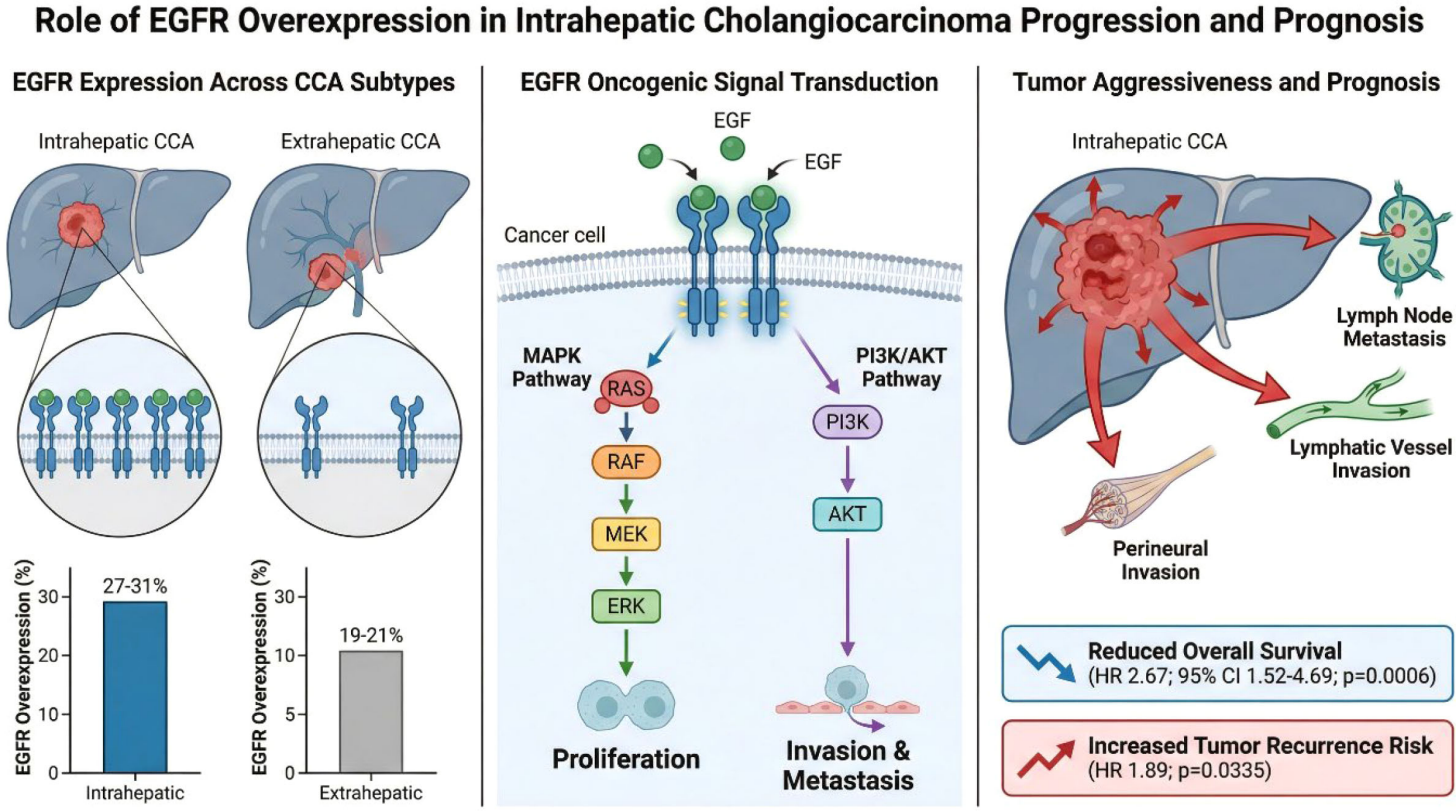
EGFR overexpression in intrahepatic cholangiocarcinoma (iCCA). EGFR activation drives MAPK and PI3K/AKT signalling, promoting proliferation, invasion, and metastasis. Clinically, EGFR overexpression is associated with lymph node metastasis, aggressive pathological features, reduced overall survival, and increased recurrence risk.

Epidermal growth factor receptor (EGFR) is overexpressed in 19-31% of CCA cases, with higher expression in intrahepatic (27-31%) compared to extrahepatic (19-21%) subtypes (49–51). EGFR functions as an oncogenic receptor tyrosine kinase that drives tumour proliferation, invasion, and metastatic progression through activation of MAPK and PI3K/AKT signalling pathways (**Figure 3**) (41, 49, 52, 53). EGFR overexpression correlates with aggressive clinicopathological features including lymph node metastasis (p=0.0006), advanced tumour stage, lymphatic vessel invasion, and perineural invasion, and serves as an independent prognostic factor for reduced overall survival (HR 2.67, 95% CI 1.52-4.69, p=0.0006) and increased tumour recurrence risk (HR 1.89, p=0.0335) in intrahepatic cholangiocarcinoma (49, 51, 54, 55).

Early-phase clinical translation of EGFR-directed CAR-T cell therapy has demonstrated proof-of-concept activity in advanced biliary tract cancers. Guo et al. reported a Phase I trial (NCT01869166) (32) of EGFR-CAR-T cells in 19 patients (14 cholangiocarcinoma, 5 gallbladder) with EGFR-positive (>50%) advanced disease (56). Following conditioning chemotherapy with nab-paclitaxel and cyclophosphamide, patients received CAR-T cell infusions at median dose 2.65×10^6^;/kg. Among 17 evaluable patients, one achieved complete response and ten achieved stable disease, with median progression-free survival of 4 months (57). Treatment was generally well-tolerated, though grade ≥3 acute fever/chills occurred in 3 patients, alongside manageable mucosal/cutaneous toxicities and pulmonary oedema (57). As previously mentioned in section 2.3, Feng et al. reported on a sequential EGFR- and CD133-directed CAR-T therapy in a single patient with metastatic cholangiocarcinoma, achieving 8.5-month and 4.5-month partial responses respectively, though with significant epidermal and vascular toxicities requiring further investigation (58).

#### 2.4.1 Clinical relevance in CCA

EGFR expression identifies a biologically aggressive subset of CCA with significantly worse prognosis following standard therapy. The dual role of EGFR as both a proliferative driver and validated immunotherapeutic target provides strong biological rationale for continued clinical development, particularly in combinatorial strategies addressing tumour heterogeneity and resistance mechanisms. However, current evidence remains limited to early-phase trials, and larger studies are needed to establish clinical efficacy and optimal patient selection criteria.

### 2.5 HER2: anatomically stratified expression and emerging therapeutic target

HER2 expression in CCA demonstrates marked anatomical heterogeneity, with significantly higher overexpression rates in extrahepatic subtypes (17–20%) compared to intrahepatic CCA (approximately 1–5%, p=0.0049) (49, 59). HER2 positivity is particularly enriched in gallbladder carcinoma and intraductal papillary neoplasms with invasive components, reflecting distinct molecular pathogenesis across biliary tract subtypes (60, 61). Clinically, HER2 overexpression correlates with adverse prognostic features and independently predicts worse survival (HR 3.08, p=0.01) (62), with shorter progression-free survival on standard chemotherapy (5.1 vs 7.4 months, p<0.001) (60). Early translational efforts have demonstrated feasibility of HER2-targeted CAR-T therapy, with Phase I data showing partial responses and disease stabilization in advanced biliary cancers (63). More recently, the antibody–drug conjugate trastuzumab deruxtecan has shown encouraging activity in HER2-positive biliary tract cancers, achieving a 36% objective response rate in the HERB trial (64).

#### 2.5.1 Clinical relevance in CCA

HER2 expression defines a molecularly distinct and anatomically stratified subset of cholangiocarcinoma associated with inferior outcomes on conventional therapy. The higher prevalence in extrahepatic disease has important implications for precision patient selection. While HER2-directed CAR-T therapy demonstrates early feasibility, antibody–drug conjugates and emerging bispecific platforms may offer improved therapeutic indices. Expansion of the HER2-low category further broadens the actionable population, though standardized HER2 testing across biliary subtypes remains essential for optimal clinical trial stratification.

### 2.6 CLDN18.2: a tight junction protein with emerging therapeutic potential

Claudin-18 isoform 2 (CLDN18.2) is a tight junction protein normally restricted to differentiated gastric epithelial cells but aberrantly expressed in a subset of CCA cases (65–68). While overall expression rates are modest (5–13% of CCA cases meeting the ≥75% moderate-to-strong threshold used in gastric cancer trials) (65, 66, 69), expression shows marked anatomical heterogeneity, with significantly higher rates in extrahepatic subtypes: perihilar CCA (22–27%) (65, 69), distal CCA (16–18%) (66, 69), and gallbladder carcinoma (16–63%) (65, 68), compared to intrahepatic CCA (2–7%) (65, 68, 69). The restricted tissue distribution of CLDN18.2 in normal tissues and its surface accessibility following malignant transformation make it an attractive therapeutic target (67), supported by clinical validation in gastric cancer where the monoclonal antibody zolbetuximab recently gained FDA approval (70). Early-phase CAR-T trials targeting CLDN18.2 have demonstrated proof-of-concept efficacy in gastric and pancreatic cancers, with manageable on-target, off-tumour toxicity primarily limited to low-grade gastritis (71). A Phase I/II trial (NCT04404595) is evaluating CLDN18.2-directed CAR-T cells (CT041) in patients with advanced digestive system cancers, including biliary tract cancers (72).

#### 2.6.1 Clinical relevance in CCA

CLDN18.2 expression defines an anatomically stratified subset of CCA, with higher prevalence in extrahepatic and perihilar locations (65–67, 69). In intrahepatic CCA, CLDN18.2 positivity has been identified as an independent adverse prognostic factor (HR 2.56, 95% CI 1.25–5.22, p=0.01) associated with reduced CD8+ T-cell infiltration and early recurrence, suggesting a biologically aggressive, immune-excluded phenotype (73). The low overall prevalence (5–13%) necessitates routine CLDN18.2 screening to identify candidates for emerging targeted therapies, including monoclonal antibodies, antibody-drug conjugates, and cellular immunotherapies (68, 70). Standardized immunohistochemical testing using validated assays (VENTANA CLDN18 43-14A) with the established ≥75% moderate-to-strong staining threshold is essential for patient selection (65, 66, 69, 70). The potential for synergistic benefit when combining CLDN18.2-targeted therapy with immune checkpoint blockade or strategies to reverse CD8+ T-cell exclusion warrants investigation in CLDN18.2-positive CCA (73).

## 3 Mechanisms of resistance to CAR therapy in CCA

**Figure 4:**
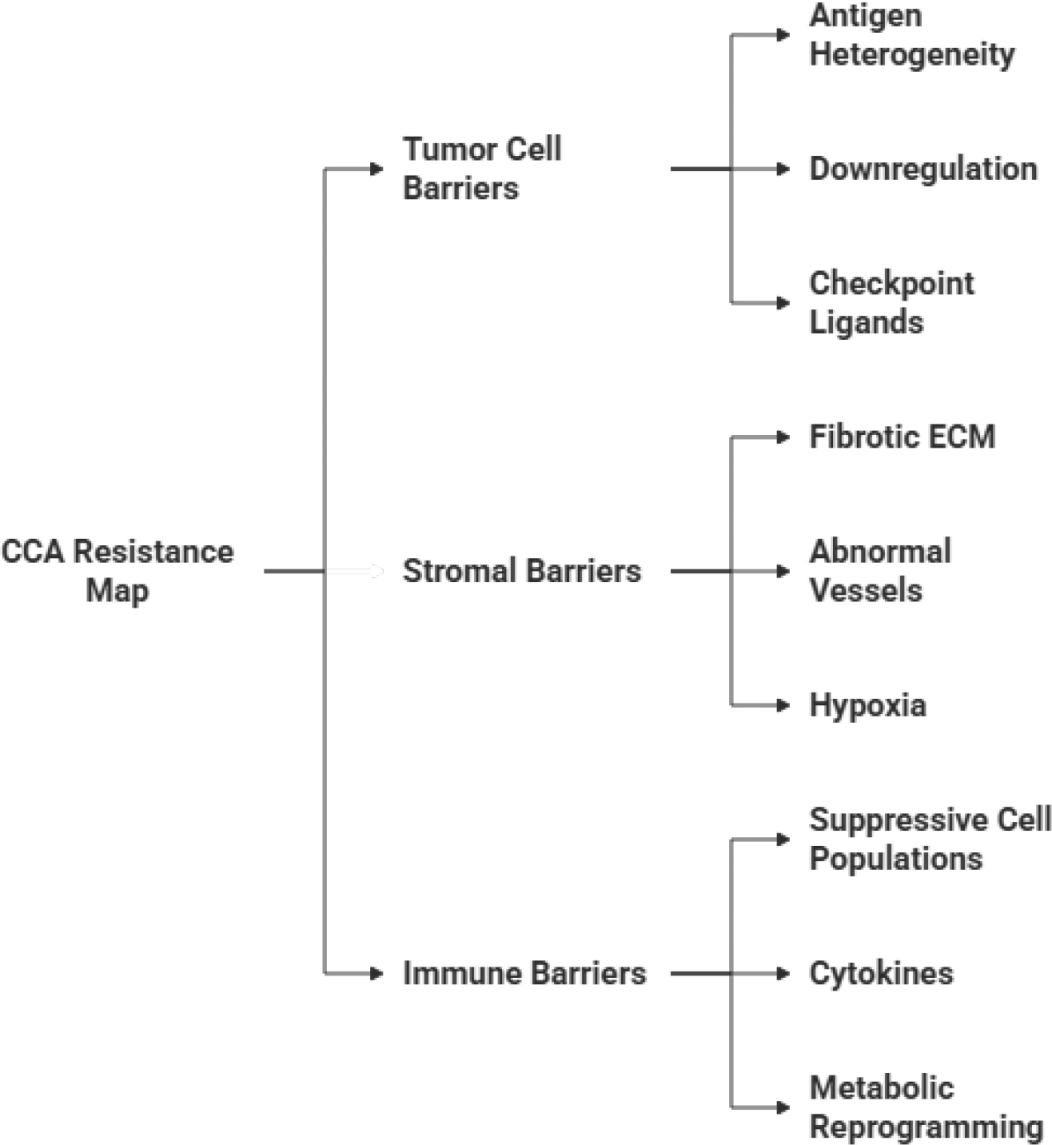
Multifactorial resistance map of cholangiocarcinoma to CAR-T cell therapy. The figure delineates the interrelated tumour cell, stromal, and immune components that collectively shape therapeutic resistance in CCA. Three interrelated resistance levels collectively limit CAR-T cell efficacy in CCA. At the tumour cell level, antigen escape via downregulation or loss of surface antigens, coupled with intrinsic resistance to T-cell-mediated killing, enables immune evasion. At the stromal level, the dense desmoplastic matrix physically restricts CAR-T cell infiltration, while CAFs actively secrete immunosuppressive cytokines (TGF-β, IL-6), chemokines (CXCL12, CCL2), and pro-angiogenic factors (VEGF) that disorganise vasculature and misdirect immune chemotaxis. At the immune system level, suppressive populations - MDSCs, M2-polarised TAMs, and Tregs - induce CAR-T cell exhaustion through checkpoint upregulation (PD-L1, TIM-3, LAG-3) and metabolic competition. The convergence of these barriers underscores the need for multimodal strategies to achieve meaningful therapeutic responses in CCA.

The clinical efficacy of CAR-based therapies in CCA is fundamentally limited by a “resistance map” of barriers operating at the level of the tumour cell, the stroma, and the immune system (**Figure 4**) (17, 22, 74, 75). These include molecular mechanisms of immune evasion, mechanical and hypoxic barriers imposed by the stroma, and the recruitment of immunosuppressive cell subsets that remodel the metabolic and cytokine milieu-highlighting the need for multimodal approaches such as stroma modulation or local ablation (e.g., histotripsy) to enhance CAR-T efficacy.

At the level of the tumour cell, resistance primarily involves mechanisms of antigen escape (10, 76, 77), where malignant cells evade detection by downregulating or losing the targeted surface antigen (a phenomenon often seen due to genetic or epigenetic changes), and through the inherent resistance of cancer cells to T-cell-mediated killing (78–80). The dense, desmoplastic stroma, a defining feature of CCA, acts as a significant physical and functional barrier (81, 82). It’s this feature, dense connective tissue, that not only restricts CAR T-cell trafficking and infiltration into the tumour core, but the tumour matrix also actively secretes immunosuppressive and pro-fibrotic factors, such as Transforming Growth Factor-beta (TGF-β) (81–89).

**Figure 5:**
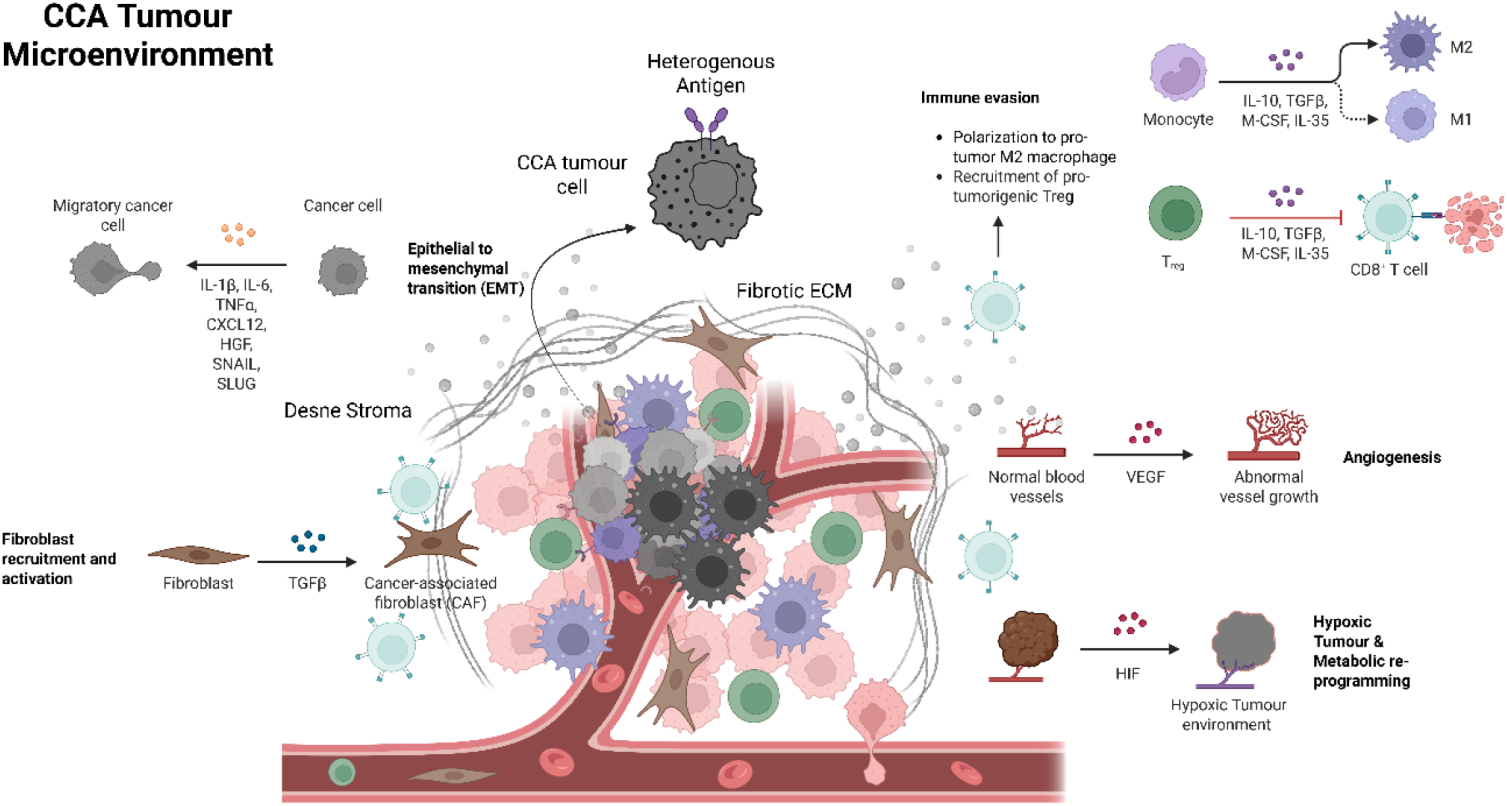
Tumour Microenvironment (TME) – This illustrates the complex and immunosuppressive microenvironment characteristic of CCA. Tumour cells display heterogeneous antigen expression and undergo epithelial–mesenchymal transition (EMT) under the influence of cytokines and growth factors such as IL-1β, IL-6, TNF-α, CXCL12, HGF, SNAIL, and SLUG, promoting migratory and invasive phenotypes. Cancer-associated fibroblasts (CAFs), activated primarily by TGF-β, secrete extracellular matrix (ECM) components that contribute to a dense fibrotic stroma and physical immune exclusion. Monocytes polarize into M2-like macrophages under the influence of IL-10, TGF-β, M-CSF, and IL-35, while Tregs reinforce immune evasion by suppressing cytotoxic CD8^+^ T-cell function through similar mediators. Angiogenic signalling via VEGF leads to aberrant vasculature, and hypoxia-induced HIF activation drives metabolic reprogramming, further sustaining tumour progression and resistance to immunotherapy.

Finally, the immune system resistance is driven by the highly suppressive tumour microenvironment (TME) (**Figure 5**) (10). Eventually the environment of the CCA is replete with inhibitory cellular components, including Myeloid-Derived Suppressor Cells (MDSCs), Tumour-Associated Macrophages (TAMs), and Regulatory T cells (T*_reg_s*), which collectively induce CAR T-cell exhaustion and anergy, thereby preventing durable anti-tumour persistence and limiting clinical response (12, 30, 78, 90–94). Addressing the multifaceted resistance map is essential for developing next-generation CAR-based strategies that can succeed against CCA.

### 3.1 Physical barriers: the desmoplastic stroma

CCA is characterized by a substantial desmoplastic TME, composed of a dense ECM, cancer associated fibroblasts (CAFs), and other stromal cells (83, 94–97). The fibrotic milieu creates a formidable barrier that physically impedes CAR-T and CAR-NK cell trafficking and infiltration into the tumour core, effectively creating an “immune desert” and preventing effector cells from reaching their targets (98, 99).

**Figure 6:**
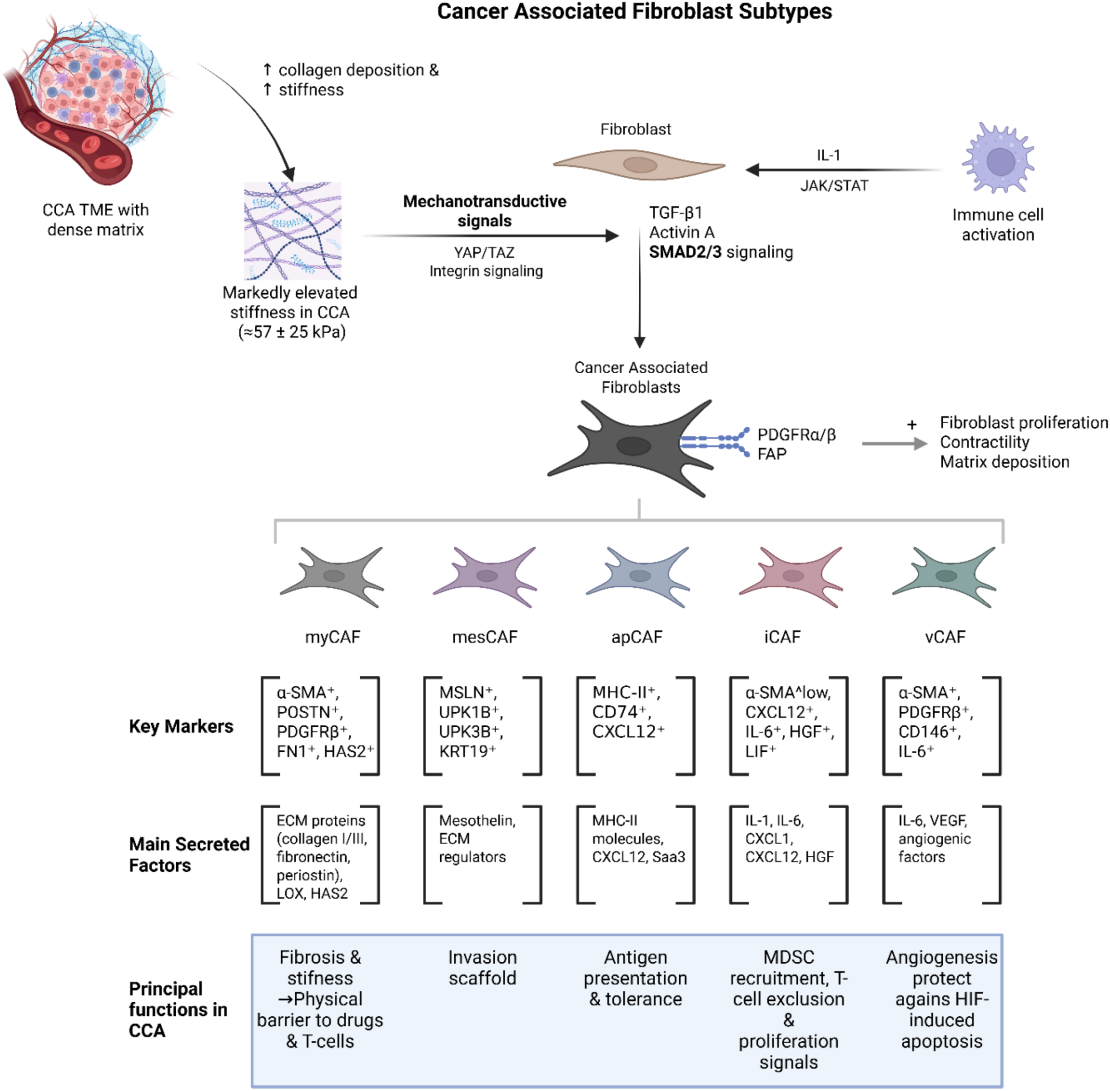
Cancer Associated Fibroblasts (CAF) in CCA – above illustrates the origin, activation pathways, and phenotypic diversity of CAFs in CCA. Fibroblasts are activated through mechanotransducive signalling (YAP/TAZ, integrins) and cytokine pathways (TGF-β1, Activin A, SMAD2/3), leading to increased matrix deposition, contractility, and stromal stiffness. Additional stimulation by IL-1 via the JAK/STAT axis promotes immune modulation. Five major CAF subtypes are shown: myCAF (α-SMA^+^, POSTN^+^, PDGFRβ^+^) responsible for ECM remodelling and fibrosis; mesCAF (MSLN^+^, UPK1B^+^, KRT19^+^) providing invasion scaffolds; apCAF (MHC-II^+^, CD74^+^, CXCL12^+^) facilitating antigen presentation and immune tolerance; iCAF (α-SMA^low, IL-6^+^, CXCL12^+^) driving myeloid recruitment and immunosuppression; and vCAF (PDGFRβ^+^, CD146^+^) promoting angiogenesis and protection from hypoxia-induced apoptosis. Collectively, these CAF subsets reinforce CCA progression through enhanced fibrosis, immune exclusion, and metabolic adaptation within the dense stromal niche.

The ECM network, rich in collagen type I, fibronectin, and laminin, forms a mechanically rigid scaffold that increases interstitial fluid pressure and restricts lymphocyte trafficking (14, 30, 98). CAFs (**Figure 6**), which dominate the stroma, secrete TGF-β (100–102), PDGF (103–106), and LOX family enzymes (107–111) that further cross-link collagen fibres, exacerbating fibrosis and promoting tumour progression (16). The increased ECM stiffness enhances integrin signalling and focal adhesion kinase (FAK) activation in tumour cells, driving proliferation, epithelial–mesenchymal transition (EMT), and invasive behaviour. The dense desmoplastic barrier not only restricts immune infiltration but also hinders drug perfusion, thereby contributing to multi-modal therapy resistance.

Mechanistically, CAFs interact with TAMs and endothelial cells, secreting CXCL12 and VEGF, which disorganize tumour vasculature and misdirect immune cell chemotaxis (16, 94, 112–117). Critically, the immunosuppressive functions of CAFs in CCA extend well beyond their role as physical barriers. In iCCA, FAP-expressing CAFs activate STAT3 signalling, which drives the production and secretion of CCL2-the principal monocyte chemoattractant in the CCA microenvironment (118). FAP+ CAFs are the primary source of CCL2 in human iCCA tissue, and this chemokine recruits CCR2-expressing MDSCs into the tumour stroma, where they suppress cytotoxic CD8^+^ T-cell responses and promote angiogenesis (118, 119). Depletion of MDSCs or blockade of the FAP/STAT3/CCL2 axis abrogates the tumour-promoting effects of CAFs in preclinical iCCA models, confirming that the pro-tumour function of fibroblastic FAP operates primarily through immunosuppressive myeloid cell recruitment rather than direct effects on tumour cell proliferation (119). Furthermore, FAP+ CAFs in iCCA secrete IL-6 and IL-33, which act on MDSCs to trigger STAT3-mediated hyperactivation of 5-lipoxygenase, resulting in leukotriene B4 release that sustains cancer stemness properties in iCCA cells (120). This CAF–MDSC–stemness axis represents a mechanistically distinct pathway through which fibroblasts indirectly promote therapeutic resistance and tumour recurrence. Beyond myeloid cell recruitment, CAFs in CCA also contribute to immunosuppression through direct modulation of immune checkpoint signalling. Conditioned medium from activated hepatic stellate cells-a major CAF precursor in CCA-stimulates macrophage differentiation through IL-6 and TGF-β production, while FAP+ CAFs simultaneously decrease the frequency of IFN-γ-producing CD8^+^ T cells within the tumour (99, 121). More recently, bile acids-which are present at exceptionally high concentrations in the biliary microenvironment-have been shown to activate GPBAR1 specifically on CCA-associated CAFs, inducing CXCL10 expression that recruits immunosuppressive neutrophils and promotes epithelial–mesenchymal transition. Notably, single-cell RNA sequencing demonstrated that GPBAR1 expression on CAFs is unique to CCA among the cancer types examined, and inhibition of the GPBAR1–CXCL10 axis enhanced the efficacy of anti-PD-1 therapy in multiple preclinical CCA models (122). Collectively, these findings establish that CAFs in CCA function as active orchestrators of an immunosuppressive niche through at least three convergent mechanisms: MDSC recruitment via CCL2, macrophage polarisation via IL-6/TGF-β, and neutrophil recruitment via bile acid–GPBAR1–CXCL10 signalling-each of which directly impairs the anti-tumour immune response and has implications for the efficacy of adoptive cellular immunotherapies.

Presence of aberrant vasculature contributes to hypoxia, a hallmark of CCA, which in turn upregulates HIF-1α, which is stabilized under these conditions, driving transcription of genes that promote angiogenesis (VEGF, PDGF) and fibrogenesis, while simultaneously promotes immune checkpoint ligand expression (e.g., PD-L1). Together, these events compound immune exclusion and CAR-T inefficacy (123–126).

Recent translational studies have identified several strategies to mitigate stromal barriers. For example, FAP-targeted CAR-T therapy, which depletes CAFs, has been shown to reduce matrix rigidity and enhance T-cell infiltration in preclinical models (127). Similarly, enzymatic desmoplasia modulation - via hyaluronidase or collagenase - has shown promise in enhancing CAR-T penetration into dense CCA tissues (128). Matrix metalloproteinases (MMPs) play dual roles in cancer (129–131), with broad-spectrum inhibitors failing in trials due to suppression of anti-tumour MMPs (129, 130) and disruption of immune cell trafficking (132), necessitating selective targeting approaches (133).

Therefore, the desmoplastic stroma represents not just a passive fibrotic structure but an active immunoregulatory component of the CCA microenvironment. Overcoming this barrier will likely require dual-targeted strategies-those that degrade or reprogram the ECM while enhancing CAR-T/NK cell trafficking, potentially transforming CCA from an “immune desert”/”Cold tumours” into an “immune-reactive”/”hot tumours” tumour.

### 3.2 Immunological barriers: checkpoints, suppressive cells, and cytokines

Even if CAR-T/NK cells successfully infiltrate the tumour, they encounter a profoundly immunosuppressive environment (30, 134–137). Within CCA, immunosuppression is orchestrated through a complex interplay of immune checkpoint pathways, suppressive immune cell subsets, and inhibitory cytokine networks that collectively induce CAR-T/NK cell exhaustion and anergy.

#### 3.2.1 Immune checkpoints

**Figure 7:**
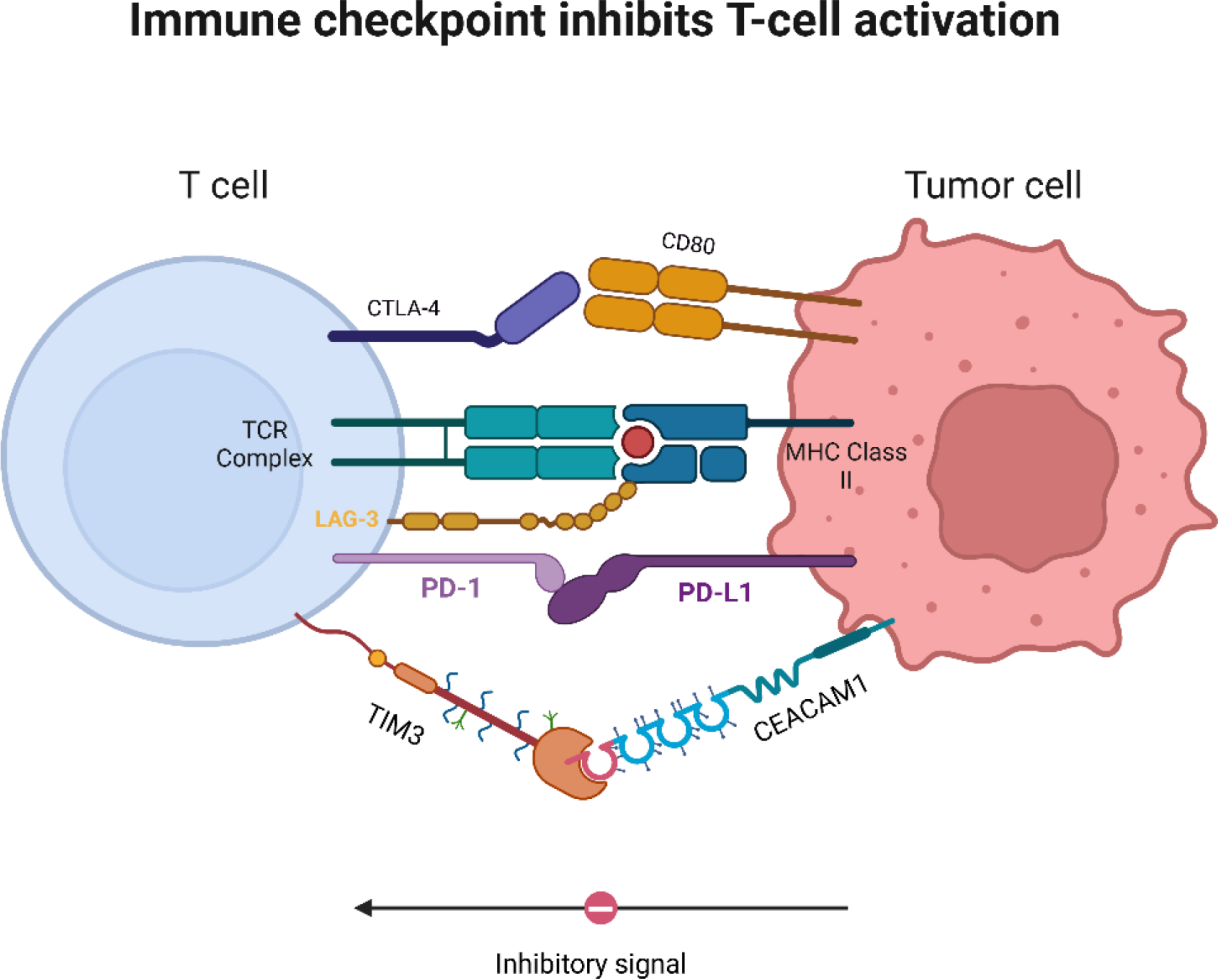
Immune checkpoints suppress T cell function. Immune checkpoint receptors expressed on T cells - including CTLA-4, PD-1, LAG-3, and TIM-3 - interact with their corresponding ligands (CD80, PD-L1, MHC class II, and CEACAM1) on tumour cells or antigen-presenting cells, delivering inhibitory signals that suppress T-cell receptor (TCR)–mediated activation. TCR – T-cell receptor; CTLA-4 – Cytotoxic T-lymphocyte–associated protein 4; PD-1 – Programmed cell death protein 1; PD-L1 – Programmed death-ligand 1; LAG-3 – Lymphocyte activation gene 3; TIM-3 – T-cell immunoglobulin and mucin-domain containing-3; CD80 – Cluster of differentiation 80 (B7-1); MHC Class II – Major histocompatibility complex class II; CEACAM1 – Carcinoembryonic antigen–related cell adhesion molecule 1.

A defining feature of the immunosuppressive TME in CCA is the upregulation of multiple immune checkpoint pathways that act in concert to paralyze effector T cell and CAR-T cell function (138–140). The coordinated expression of PD-1/PD-L1 (141–147), CTLA-4 (148, 149), TIM-3 (78, 150–153), LAG-3 (91, 154, 155), and TIGIT (90, 156–158) establishes a state of immune exhaustion characterized by diminished cytokine release, impaired cytotoxic granule exocytosis, and metabolic dysfunction in tumour-infiltrating lymphocytes (TILs) (**Figure 7**). Additionally, the inhibitory interactions serve as molecular “brakes” on the immune system, enabling tumour cells to evade immune destruction and facilitating the persistence of a tolerogenic microenvironment.

#### 3.2.2 Suppressive cytokines

The TME is saturated with soluble immunosuppressive factors, most notably TGF-β, IL-10, and IL-6, which directly inhibit T cell and NK cell activation, proliferation, and cytotoxic function.

TGF-β plays a dominant role in the TME (100, 159). It suppresses T-cell receptor (TCR) signalling, inhibits cytotoxic granule release, and drives CD4^+^ T-cell differentiation into FoxP3^+^ Tregs, thereby amplifying the immunosuppressive cell population within the tumour (160–162). Moreover, TGF-β contributes to fibrosis and extracellular matrix remodelling, reinforcing the desmoplastic stroma that physically restricts immune cell infiltration. In CAR-T therapy, TGF-β activates SMAD2/3 signalling, inducing exhaustion and apoptosis that limit CAR-T persistence (162–164). To overcome this, next-generation “armoured CARs” have incorporated dominant-negative TGF-β receptors (dnTGFβRII) to maintain effector function even in TGF-β–rich environments (165, 166).

IL-10, another major immunosuppressive cytokine in CCA, inhibits antigen-presenting cell (APC) function by downregulating MHC-II and co-stimulatory molecules, thereby limiting CAR-T/NK-cell priming and reducing IFN-γ and IL-2 production (92, 167–169). Sustained IL-10 signalling also polarizes macrophages toward an M2-like phenotype, promoting angiogenesis, tumour growth, and continued cytokine-mediated suppression (170).

Meanwhile, IL-6-secreted by tumour and stromal cells-drives tumour-promoting inflammation via JAK/STAT3 activation, which enhances PD-L1 expression, T-cell exhaustion, and resistance to apoptosis (171–175). Chronic IL-6/STAT3 activity also impairs dendritic cell maturation, further disrupting antigen presentation (17, 176, 177). Elevated IL-6 levels correlate with poor prognosis and reduced immunotherapy response in CCA and other solid tumours (174, 178, 179).

Together, the complex cytokine-driven network sustains a metabolically and immunologically hostile TME, impairing CAR-T and CAR-NK recruitment and persistence (180, 181). Current therapeutic strategies-such as TGF-β inhibitors (182, 183), IL-6R blockade, and IL-10 antagonists, or cytokine-resistant CAR designs (78, 184–186)-aim to neutralize these pathways and reprogram the CCA microenvironment from an immunologically inert “cold” tumour into one that supports robust, sustained cytotoxic immune activity.

#### 3.2.3 Suppressive cell populations

The TME is densely populated with myeloid-derived suppressor cells (MDSCs) and tumour-associated macrophages (TAMs), which further contribute to immune suppression and hinder CAR therapy efficacy (186, 187).

Beyond their sheer abundance, MDSCs and TAMs actively sculpt an immunosuppressive ecosystem that impedes CAR cell persistence and cytotoxicity (189–191). In CCA, tumour-derived cytokines such as GM-CSF, IL-6, and VEGF drive the recruitment and expansion of these myeloid populations (10, 30, 192). Once established, MDSCs release arginase-1, nitric oxide, and reactive oxygen species, which disrupt T-cell receptor (TCR) signalling and suppress CAR-T activation (193–196). Meanwhile, TAMs-particularly those polarized toward the M2 phenotype-secrete IL-10, TGF-β, and VEGF, reinforcing immune tolerance, angiogenesis, and fibrosis (197–199). This crosstalk not only shields tumour cells from immune attack but also supports the desmoplastic stroma that physically restricts CAR-T and CAR-NK infiltration. Therapeutic strategies targeting these myeloid compartments-such as CSF1R inhibitors (200), CCR2 blockade, or reprogramming agents that repolarize TAMs toward an M1 phenotype (201)-are being explored to dismantle these suppressive barriers and enhance the efficacy of adoptive cellular immunotherapies in CCA.

### 3.3 Tumour-intrinsic barriers: antigen heterogeneity and metabolism

The tumour cells themselves employ resistance strategies.

#### 3.3.1 Antigen heterogeneity

**Figure 8:**
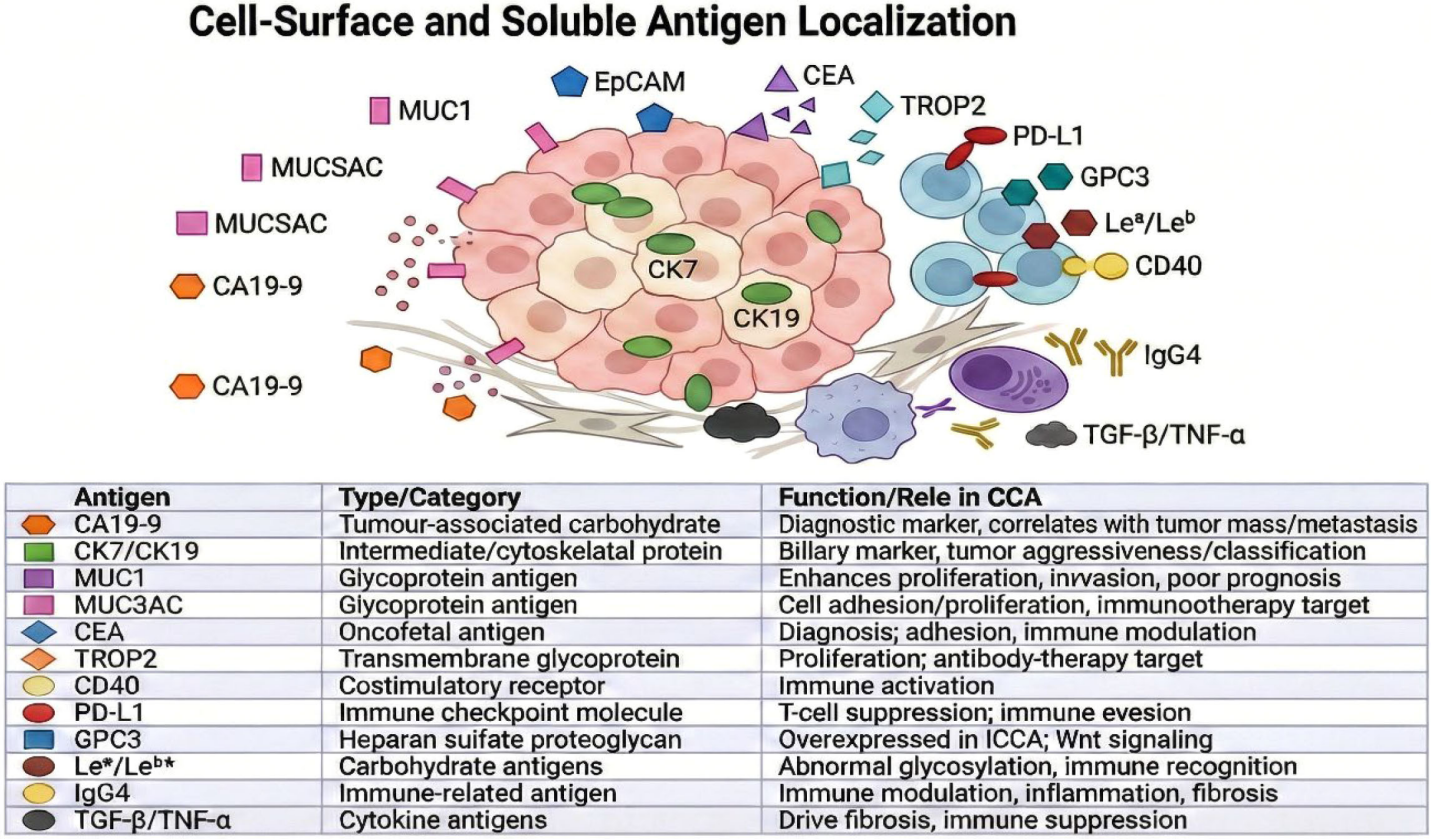
Heterogenous antigen presentation in CCA (204–212).

Antigen heterogeneity poses a major barrier to durable CAR-T efficacy in cholangiocarcinoma (CCA) (14, 188). Even within a single lesion, spatial and temporal variation in antigen expression-driven by genetic instability, epigenetic modulation, and selective immune pressure-can lead to antigen loss variants that evade CAR recognition (202, 203). Moreover, CCA frequently co-expresses multiple tumour-associated antigens (**Figure 8**) at varying densities, potentially limiting the depth and durability of response to single-antigen CAR strategies (14). To counter this, next-generation strategies such as dual- or tandem-CARs, logic-gated CAR circuits, and multi-targeted NK or bispecific constructs are being developed to broaden antigen coverage, reduce escape, and enhance therapeutic persistence in heterogeneous CCA tumours (213–217).

#### 3.3.2 Metabolic barriers

The dense and poorly vascularized TME is often hypoxic and nutrient deprived. Tumour cells outcompete immune cells for essential nutrients like glucose, creating a metabolically hostile environment that impairs the function and survival of infiltrating CAR cells (218). CCA cells engage in aerobic glycolysis (the Warburg effect), depleting glucose and producing lactate, which acidifies the TME and inhibits CAR-T and NK cell proliferation and cytokine secretion (181, 219, 220). High levels of adenosine, generated by ectoenzymes CD39/CD73, further suppress immune cell activation and survival (221–224). Together, these metabolic stressors form a “nutrient competition zone” that favours tumour growth over immune effector persistence (220, 225). Targeting metabolic checkpoints-such as HIF-1α, adenosine signalling, or lactate metabolism-or reprogramming CARs with enhanced oxidative metabolism may offer promising strategies to restore CAR functionality and durability in the metabolically hostile CCA microenvironment.

## 4 Next-generation engineering strategies to overcome resistance

Counteracting this resistance map requires a sophisticated engineering pipeline, moving from novel target discovery to the creation of multi-functional, armoured CAR constructs (226–228).

### 4.1 Advanced antigen discovery pipelines

The identification of better TAAs is paramount. Modern omics pipelines are accelerating this process (229–232). Single-cell RNA-sequencing and spatial proteomics allow for high-resolution profiling of individual tumour cells, enabling the discovery of antigens that are consistently expressed on cancer cells but absent from critical healthy tissues (233, 234). Spatial transcriptomics adds another layer of insight by mapping antigen expression within the TME to understand heterogeneity and identify targets present on both tumour cells and supportive stromal components.

### 4.2 Armoured CARs to counteract immunosuppression

To survive the TME, CARs must be “armoured.” This includes (235, 236):

#### 4.2.1 Checkpoint-resistant CARs

These are CARs engineered to be resistant to inhibitory signals (186, 237, 238). A leading example is the sextuplet-knockdown CAR-T cell, where shRNAs are used to simultaneously downregulate PD-1, TIM-3, TIGIT, and the receptors for TGF-β, IL-10, and IL-6 (78). This strategy has been shown to dramatically enhance anti-tumour activity and partially improve persistence in preclinical CCA models.

#### 4.2.2 Cytokine-secreting CARs

**Figure 9:**
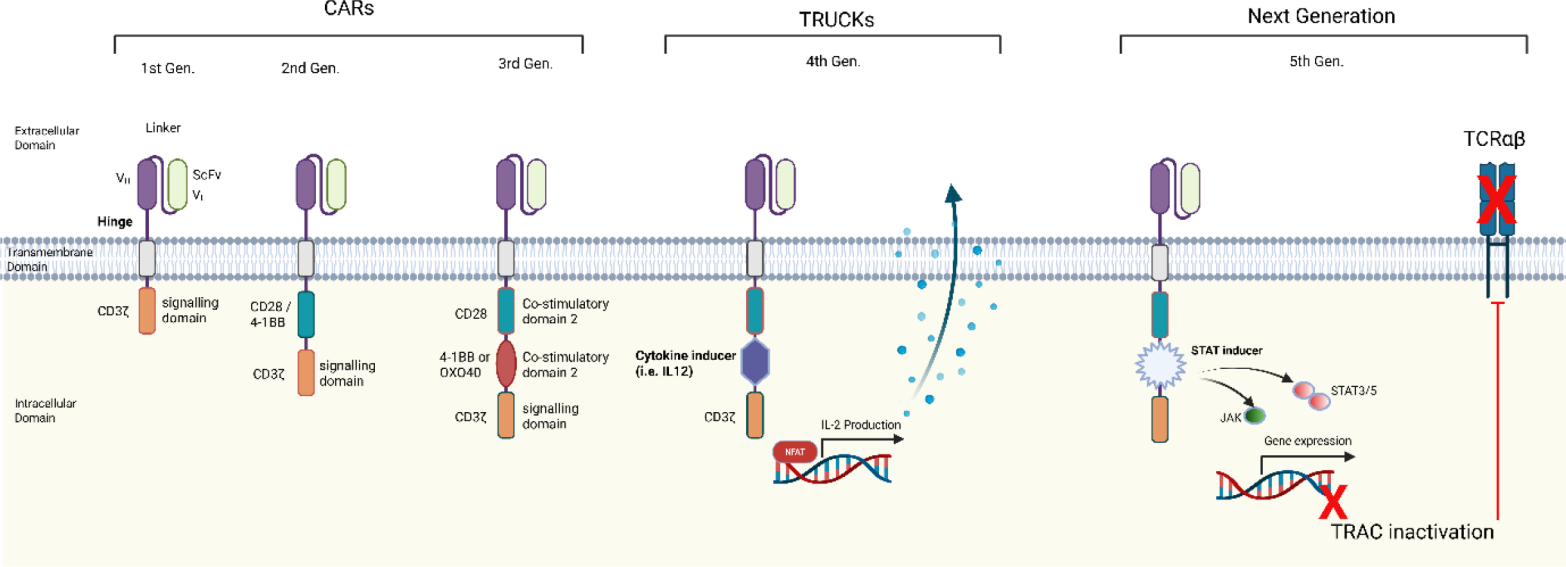
Overview of CAR-T cell construct generations: First generation CARs featured only the CD3ζ signalling domain, resulting in limited expansion and persistence. Second generation CARs added a costimulatory signal (CD28 or 4-1BB) for better cytotoxicity and longevity. Third generation CARs included two costimulatory domains to further boost T-cell proliferation and survival. Fourth generation CARs (TRUCKs) incorporated domains that induce cytokine production upon antigen recognition, enhancing immune modulation. Fifth generation CARs, based on second generation designs with gene editing to remove TCR chains and add a STAT3-binding IL-2 receptor β-chain, provide three synergistic activation signals, driving robust T-cell function. [image adapted from (240)].

Known as “T cells redirected for universal cytokine-initiated killing” (TRUCKs) (**Figure 9**), these CARs are engineered to secrete pro-inflammatory cytokines like IL-12 upon antigen engagement, helping to remodel the TME and recruit a broader endogenous immune response (239, 241–243).

### 4.3 Multi-antigen targeting platforms

To combat antigen escape, platforms targeting multiple antigens are crucial. This has been tested clinically via a “cocktail” infusion of EGFR- and CD133-specific CAR-T cells (58). More advanced designs include dual-CARs (dCAR-T), which express two distinct scFvs to recognize two different antigens simultaneously (244). Next-generation synNotch circuits offer a logic-gated approach, where recognition of a first antigen primes the CAR-T cell to then recognize and kill cells expressing a second, more tumour-specific antigen, thereby enhancing both efficacy and safety (245, 246).

## 5 Synergistic adjuvant therapies

CAR cell monotherapy is unlikely to succeed in isolation against a deeply entrenched solid tumour. A key future direction is the use of rational adjuvant therapies to prime the tumour for immune attack (247–250). **Table 3** summarizes potential adjuvant therapies to augment CAR therapy.

**Table 2:** Completed clinical trials in cholangiocarcinoma.

Trial ID	Phase	Status	Target antigen	Patient cohort	Key efficacy outcomes	Key safety outcomes	Reference
NCT01869166	Phase I	**Completed**	EGFR CAR-T	**n=14 CCA** n=5 GBC (19 BTC total) 17 evaluable	• CR: 1/17 (5.9%) • SD: 10/17 (58.8%) • **mPFS: 4 months** (range 2.5-22) • Tcm enrichment predicted better outcomes	• Grade ≥3 AEs: 3/19 (fever/chills) • Grade 1-2 CRS (manageable) • Grade 1-2 skin/mucosal toxicity • No treatment-related deaths	Guo Y, et al., 2018 (32, 56)
NCT01869166 + NCT02541370	**Case Report**	Published 2017	**Sequential** EGFR→CD133	**n=1** 52F advanced metastatic CCA Failed chemo/RT	• **EGFR CAR-T**: 8.5-month PR (>80% shrinkage) • **CD133 CAR-T**: 4.5-month PR after EGFR resistance • Total duration: 13 months	• **EGFR**: Grade 1-2 rash, manageable • **CD133**: Grade 3-4 rash, ascites, pleural effusion • Required methylprednisolone + anti-TNF • **On-target, off-tumour toxicity** from CD133 on normal epithelium/endothelium	Feng KC, et al., 2017 (31, 32, 58)
NCT01935843	Phase I	**Completed**	HER2 CAR-T	**n=4 pCCA** **n=4 iCCA** (Total n=8 CCA; 11 total with 3 pancreatic)	• PR: 1/11 (9.1%) - **4.5 months PFS** • SD: 3/8 CCA patients • PD: 4/8 CCA patients • **Median PFS: 3.25 months** (range 1.5-5) • Median dose: 2.45 × 10^6^;/kg	• Grade 3 acute febrile illness: 1 patient • Grade 3 transaminase abnormality: 1 patient • Mild-moderate fatigue, nausea, myalgia • Lymphopenia (conditioning-related) • **Well-tolerated overall**	Feng K, et al., 2018 (63, 251)

TOTAL PATIENTS ACROSS ALL CLINICAL TRIALS: n=23 CCA patients (14 + 1 + 8).

CR, Complete Response (tumour completely disappeared); PR, Partial Response (tumour shrunk by ≥30%); SD, Stable Disease (tumour neither grew significantly nor shrunk); PD, Progressive Disease (tumour grew/worsened); mPFS, median Progression-Free Survival (average time before cancer worsened).

**Table 3:** Adjuvant classes for combination with CAR therapy in CCA.

Adjuvant class	Mechanism of action	Level of evidence (in CCA Context)	Ref
Checkpoint Inhibitors	Block inhibitory pathways (e.g., PD-1/PD-L1) to reverse T-cell exhaustion and enhance CAR cell function.	Moderate. Combination with anti-EGFR CAR-T cells has been tested clinically. Synergistic potential is high but not yet validated in large trials.	(57, 58, 186)
Natural Compounds	Inhibit key oncogenic pathways (PI3K/AKT, FGFR), induce apoptosis, and modulate the immune TME.	Preclinical/Hypothesis-generating. Thymoquinone, curcumin, and EGCG show potent anti-CCA activity in preclinical models.	(252–255, 320, 321)
Small Molecule Inhibitors	Target specific mutations (e.g., FGFRi, IDHi) or signalling pathways to debulk the tumour and reduce suppressive signalling.	High. FGFR and IDH inhibitors are approved for CCA. Combination with CAR therapy represents a hypothesis-generating strategy that requires preclinical validation in CCA	(322–325)
Stromal Disruptors	Degrade the fibrotic extracellular matrix (e.g., using enzymes like heparanase) to improve CAR cell infiltration.	Preclinical. Strategies have shown promise in pancreatic cancer models but are not yet clinically validated in CCA.	(238, 326, 327)

The use of natural compounds is a particularly novel, though preclinical, avenue. By inhibiting core survival pathways, these agents could lower the threshold for CAR-mediated apoptosis, while their immunomodulatory properties could help create a more favourable TME. Thymoquinone, curcumin, and EGCG demonstrate convergent anticancer mechanisms in preclinical CCA models through multi-pathway inhibition of PI3K/Akt, NF-κB, and STAT3 signalling, coupled with upregulation of pro-apoptotic mediators (BAX, DR4/DR5) and suppression of survival proteins (Bcl-2, XIAP, survivin) (252–254). These agents induce mitochondrial-mediated apoptosis, inhibit invasion via MMP-2/9 downregulation, and demonstrate *in vivo* tumour suppression in xenograft models (254). Curcumin exhibits particular promise through synergistic enhancement of gemcitabine efficacy via LAT2/glutamine pathway disruption (255). By targeting core resistance mechanisms, these compounds may lower the apoptotic threshold for CAR-T-mediated cytotoxicity, warranting evaluation in combination immunotherapy studies despite incomplete characterization of their tumour microenvironment immunomodulatory effects.

Additionally, CCA exhibits anatomically stratified molecular alterations: FGFR2 fusions (10-15%) and IDH1 mutations (10-20%) occur almost exclusively in intrahepatic CCA, while HER2 amplifications are enriched in extrahepatic subtypes (17-20% vs 1-5% iCCA) (256–260). FDA-approved inhibitors targeting these alterations (pemigatinib, futibatinib for FGFR2; ivosidenib for IDH1) induce meaningful tumour responses in molecularly selected patients (261–265). Rationally designed combinations integrating these targeted agents with CAR-T therapy could enhance antigen exposure through cytoreduction, modulate the immunosuppressive tumour microenvironment, and improve CAR cell persistence. Prospective evaluation of sequential or concurrent strategies remains a critical translational priority in precision CCA therapy.

## 6 Clinical translation: critical appraisal and future trial design

### 6.1 Critical appraisal of the current clinical landscape

The current clinical trial landscape for CAR therapy in CCA (**Table 2**), while promising, is marked by several weaknesses (28, 266, 267). Most studies are early-phase, single-centre trials with small sample sizes, making it difficult to draw definitive conclusions (266). A key challenge observed across multiple trials is the limited persistence of CAR-T cells *in vivo*. Furthermore, trials often suffer from inadequate patient stratification, enrolling patients without confirming high-level expression of the target antigen, which can dilute potential efficacy signals. The risk of significant toxicity, especially on-target, off-tumour effects, remains a major concern that requires better management strategies.

### 6.2 Strategic recommendations for next-generation clinical trials

To overcome these limitations, future trials should be more sophisticated:

#### 6.2.1 Biomarker-driven enrolment

Trials should mandate rigorous tumour tissue–based biomarker screening to confirm high and relatively uniform surface expression of the target antigen by immunohistochemistry, RNA profiling, or quantitative proteomics. Peripheral blood biomarkers - such as circulating tumour DNA (ctDNA) or circulating tumour cells (CTCs) - may complement tissue assessment but cannot substitute for direct antigen validation. The integration of real-time biopsies and omics analysis can guide treatment decisions and monitor for antigen loss (268–271).

#### 6.2.2 Adaptive trial designs

Given the heterogeneity of CCA, adaptive designs that allow for modifications-such as switching or adding CAR-T cell targets based on response and resistance patterns-should be implemented (272–275). Although there are no CCA specific adaptive trails, this approach would require baseline and serial assessment of target antigen expression through tumour biopsies, advanced imaging, and molecular profiling (immunohistochemistry, RNA sequencing, spatial proteomics), complemented by circulating tumour DNA analysis for clonal evolution monitoring. Predefined actionable triggers - including antigen loss, emergence of alternative antigen expression, progression in antigen-low lesions, or pathway reprogramming - would permit protocol-specified interventions such as switching CAR constructs, adding dual-antigen products, or combining checkpoint blockade or targeted therapies. Successful implementation requires rapid biomarker processing, centralized molecular review, predefined decision algorithms, and regulatory frameworks supporting modular treatment arms to enable real-time therapeutic optimization while maintaining trial integrity.

#### 6.2.3 Incorporation of safety and persistence strategies

The routine inclusion of suicide switches (e.g., iCasp9) can mitigate severe toxicities (276–278). Strategies to boost persistence, such as selecting for Tcm-rich infusion products or using CARs with optimized co-stimulatory domains (like CD27), should be prioritized (279, 280).

## 7 A roadmap for the future

**Figure 10:**
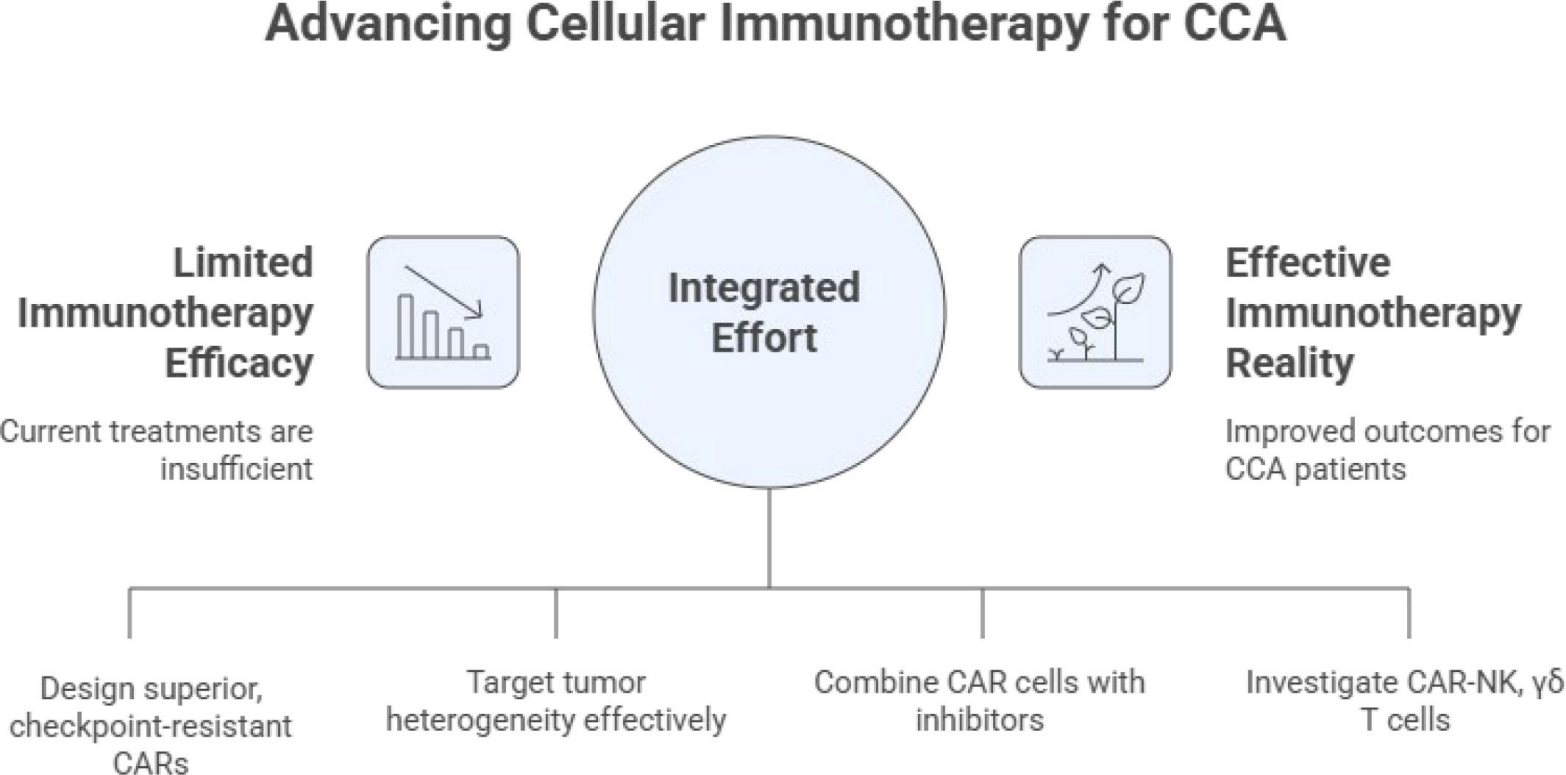
Future roadmap for advancing cellular immunotherapy for CCA. Four strategic pillars are proposed for clinical translation. Pillar 1 (Advanced CAR Engineering): checkpoint-resistant and cytokine-secreting armoured constructs, metabolic reprogramming (e.g., GLUT5-mediated fructose utilisation), and bile acid–responsive promoters for biliary-specific CAR activity. Pillar 2 (Multi-Antigen Strategies): cocktail infusions, dual-target CARs, and logic-gated systems (e.g., synNotch circuits) to address tumour heterogeneity while preserving selectivity. Pillar 3 (Synergistic Adjuvant Therapies): combination with checkpoint inhibitors, immunomodulatory compounds, targeted inhibitors (FGFRi, IDHi), and mechanical tumour disruptors to prime the TME. Pillar 4 (Alternative Effectors): diversification beyond αβ T cells to include off-the-shelf CAR-NK cells, CAR-macrophages with phagocytic and antigen-presenting capacity, and γδ T cells. Concurrent advances across all four pillars are required to overcome the multifactorial resistance landscape of CCA.

The path to making cellular immunotherapy a reality for CCA requires an integrated effort across four key pillars (**Figure 10**).

### 7.1 Pillar 1: advanced CAR engineering

Future research should prioritize armoured CARs designed for superior function. This means going beyond developing checkpoint resistant CARs, with simultaneous knockdown of multiple inhibitory receptors (e.g., PD-1, TIM-3, TGFβR), and creating cytokine-secreting CARs (TRUCKs) that release pro-inflammatory cytokines like IL-12 to remodel the TME (78, 235, 239, 242, 281).

CCA tumours often have a unique metabolic signature: they thrive in a low-glucose, high-fructose environment (282). Standard CAR T-cells rely heavily on glucose. In the CCA TME, the tumour hogs the glucose, starving the T-cells creating metabolic competition. One way to overcome this is to engineer CAR T-cells to express GLUT5 (a fructose transporter usually found in the gut/liver but not T-cells). This approach has been tested in AML (283, 284) and solid tumour models including prostate cancer (283, 285), with GLUT5-expressing CAR-Ts demonstrating superior cytotoxicity, migration, and *in vivo* tumour control compared to conventional CAR-Ts (285, 286). This approach reprograms CAR-T cell bioenergetics to exploit alternative nutrient sources within the metabolically restrictive TME, enhancing cellular persistence and anti-tumour activity. Thus, preventing exhaustion without needing to target a new surface antigen.

There is mounting evidence that bile duct and liver microbiota shape immune activation and response to immunotherapy (287). No CAR platform has yet integrated this. A hypothesis-generating approach that requires CCA-specific validation with a novel direction is to engineer CAR-T cells with Bile acid–sensitive promoters, allowing local tuning of CAR activity in the biliary tree. This turns immunotherapy into a symbiotic interface with local microbial ecology within the biliary tress.

### 7.2 Pillar 2: multi-antigen strategies

Since antigens on cancers and their corresponding, nonredundant, healthy tissues are identical, the lack of consistently expressed tumour antigens for solid organ malignancies now results in a lack of specificity (288). To overcome tumour heterogeneity, the field must clinically test multi-antigen targeting platforms. Promising strategies include cocktail infusions of CAR-T cells with different specificities and engineering single cells with dual- or tri-target CARs. Advanced, logic-gated systems like synNotch circuits could further enhance specificity by requiring the presence of two distinct antigens before triggering a full cytotoxic response (246, 289–293).

Most multi-antigen platforms assume that antigens A and B are co-expressed in the same cell. CCA contests that presumption: specific antigens are localised at invasive fronts, others adjacent to ducts, and some within hypoxic cores (294). An anticipated improvement is the development of CAR systems that can comprehend spatial antigen patterns. For instance, a logic-gated CAR that activates exclusively in the presence of one antigen on the apical membrane and another on the basolateral membrane, leveraging the polarity loss of CCA; or CARs engineered to activate solely in the presence of two antigens at designated density ratios, thus interpreting a “CCA-specific spatial signature.” This progresses beyond dual-antigen recognition to spatial antigen computation, improving selectivity against healthy biliary epithelium.

### 7.3 Pillar 3: synergistic adjuvant therapies

The next wave of clinical trials should focus on rational combination strategies. This includes combining CAR cells with systemic checkpoint inhibitors (295, 296). A particularly novel avenue is the integration of natural compounds, such as curcumin and thymoquinone, which can inhibit key oncogenic pathways, modulate the immune TME, and potentially remodel the dense tumour stroma (297, 298). Combining CAR-based therapies with mechanical tumour disruptors-such as radiofrequency ablation (RFA) or high-intensity focused ultrasound (HIFU)-represents a promising strategy to maximize therapeutic impact by enhancing tumour antigen release, immune cell infiltration, and CAR effector function (299–301).

### 7.4 Pillar 4: exploration of alternative effectors

The limitations of conventional αβ T cells necessitate the exploration of alternative immune effector cells. CAR-NK cells are a leading alternative (302, 303), offering an “off-the-shelf” allogeneic source with a potentially better safety profile. Other emerging platforms warrant investigation, such as gamma-delta (γδ) T cells and CAR-macrophages (CAR-M), which can phagocytose tumour cells and present antigens to initiate a broader adaptive immune response (304–307).

CAR-NK cells offer MHC-unrestricted tumour recognition through innate activating receptors (NKG2D, NKp30, DNAM-1) and critically do not cause GvHD, enabling allogeneic “off-the-shelf” manufacturing from cord blood, peripheral blood, or iPSC-derived sources (308, 309). The first-in-human trial of cord blood-derived CAR-NK cells confirmed safety without CRS, neurotoxicity, or GvHD (308), and preclinical biliary tract models have demonstrated NK cell cytotoxicity against CCA cells when combined with IL-2 or IL-15 (16). However, shorter *in vivo* persistence relative to CAR-T cells and TGF-β-mediated suppression within the desmoplastic CCA stroma remain key limitations (308, 310). CAR-macrophages (CAR-M) exploit natural myeloid tropism for solid tumour infiltration, combining antigen-specific phagocytosis with TME remodelling through M1 polarisation and cross-presentation of tumour antigens to adaptive immunity (311, 312). The first-in-human phase I trial of anti-HER2 CAR-M (CT-0508) demonstrated feasibility and tolerability without lymphodepletion (313), though the risk of M2 re-polarisation by CCA-derived TGF-β and limited macrophage proliferative capacity necessitate further optimisation (304, 311).

γδ T cells are uniquely suited to hepatobiliary immunotherapy, bridging innate and adaptive immunity through dual recognition via TCR-mediated phosphoantigen sensing and NKG2D-dependent stress ligand detection (MICA/B, ULBPs), both independent of MHC restriction (314, 315). Liver-resident Vδ1 T cells exhibit tissue-residency markers (CD69^+^CD49a^+^), express hepatic homing receptors CXCR6/CXCR3, and persist for over 10 years in hepatic tissue (316). CCA-specific clinical evidence is encouraging, allogeneic Vγ9Vδ2 T cell infusion achieved lymph node regression in a stage IV CCA patient (317), and a randomised trial combining locoregional ablation with γδ T cell transfer in iCCA demonstrated significantly prolonged progression-free survival (8 vs 4 months, P = 0.021) (318). Ex vivo expanded Vγ9Vδ2 T cells also demonstrate direct cytotoxicity against CCA cell lines via perforin–granzyme degranulation (319). Limitations include low circulating frequency requiring ex vivo expansion and the existence of regulatory γδ subsets that may paradoxically promote tumour progression (314, 315).

## 8 Conclusion

Cellular immunotherapy will only succeed in CCA through integration, not isolated innovation. The formidable barriers of antigen heterogeneity and a deeply immunosuppressive TME demand a multi-pronged assault. By systematically advancing research across the pillars of advanced CAR engineering, multi-antigen targeting, synergistic adjuvants, and alternative effectors, the field can overcome current limitations. The central challenge-and the future research agenda-is to integrate these innovative strategies into cohesive, biomarker-driven clinical trials. Only then can we hope to unlock the full potential of cellular immunotherapy and transform outcomes for patients with this devastating disease.
